# Treatment of pediatric convulsive status epilepticus

**DOI:** 10.3389/fneur.2023.1175370

**Published:** 2023-06-29

**Authors:** Lena-Luise Becker, Alexander Gratopp, Christine Prager, Christian E. Elger, Angela M. Kaindl

**Affiliations:** ^1^Department of Pediatric Neurology, Charité-Universitätsmedizin Berlin, Berlin, Germany; ^2^Center for Chronically Sick Children, Charité-Universitätsmedizin Berlin, Berlin, Germany; ^3^Institute of Cell Biology and Neurobiology, Charité-Universitätsmedizin Berlin, Berlin, Germany; ^4^Department of Pediatric Pneumonology, Immunology and Intensive Care, Charité-Universitätsmedizin Berlin, Berlin, Germany; ^5^Beta Clinic, Bonn, Germany

**Keywords:** status epilepticus, pediatric, treatment, epilepsy, benzodiazepine

## Abstract

Status epilepticus is one of the most common life-threatening neurological emergencies in childhood with the highest incidence in the first 5 years of life and high mortality and morbidity rates. Although it is known that a delayed treatment and a prolonged seizure can cause permanent brain damage, there is evidence that current treatments may be delayed and the medication doses administered are insufficient. Here, we summarize current knowledge on treatment of convulsive status epilepticus in childhood and propose a treatment algorithm. We performed a structured literature search *via* PubMed and ClinicalTrails.org and identified 35 prospective and retrospective studies on children <18 years comparing two and more treatment options for status epilepticus. The studies were divided into the commonly used treatment phases. As a first-line treatment, benzodiazepines buccal/rectal/intramuscular/intravenous are recommended. For status epilepticus treated with benzodiazepine refractory, no superiority of fosphenytoin, levetirazetam, or phenobarbital was identified. There is limited data on third-line treatments for refractory status epilepticus lasting >30 min. Our proposed treatment algorithm, especially for children with SE, is for in and out-of-hospital onset aids to promote the establishment and distribution of guidelines to address the treatment delay aggressively and to reduce putative permanent neuronal damage. Further studies are needed to evaluate if these algorithms decrease long-term damage and how to treat refractory status epilepticus lasting >30 min.

## Introduction

Status epilepticus (SE) refers to a prolonged clinical and/or electrographic seizure that does not cease within an expected time frame (1). SE is one of the most common life-threatening neurological emergencies in childhood, with about 17–23 episodes per 100,000 children annually and with the highest incidence in the first 5 years of life (1–9). SE can result in neurologic morbidity and has an overall mortality rate of up to 3% (1–9). Most common SE etiologies in children are prolonged febrile seizures, sudden discontinuation of anti-seizure medication (ASM) in children with treated epilepsy, acute central nervous system (CNS) insult, and chronic neurological conditions (10, 11).

The International League Against Epilepsy (ILAE) defined SE as “a condition resulting either from the failure of the mechanism responsible for seizure termination or from the initiation of mechanisms which lead to abnormally prolonged seizures (after time point t1). It is a condition that can have long-term consequences (after time point t2), including neuronal death, neuronal injury, and alteration of neuronal networks, depending on the type and duration of seizures” (12). The ILAE task force suggested treatment to be initiated at t1, and if t2 is reached, that treatment should be exacerbated to prevent long term damage (12). They further delineated t1, i.e., the time when a seizure is likely not to be self-limiting, to be 5 min in generalized tonic–clonic (GTC) SE, 10 min in focal SE with impaired awareness, and 10–15 min in absence SE, although there is no data on the latter (12, 13). Furthermore, they defined t2 to be 30 min for GTC SE and > 60 min for focal SE with impaired awareness (Table 1) (12). For other SE subtypes, including febrile seizure SE and focal SE with awareness, no duration has been proposed, leaving the older definition of >30 min (12). This earlier initiation and more aggressive therapeutic approach in the new ILAE definition is based on the knowledge that treatment becomes increasingly difficult the longer a SE lasts, in part due to receptor trafficking such as a reduction of GABA_A_ receptor-mediated inhibition (1, 3, 14).

**Table 1:** SE definition according to ILAE.

SE type	Treatment initiation (t1)	Suspected long-term consequences (t2)
Tonic–clonic SE	5 min	>30 min
Focal SE with impaired consciousness	10 min	>60 min
Absence SE	10–15 min	unknown

Treatment should be started at time point t1 as a seizure becomes unlikely to self-terminate, and treatment should be exacerbated aggressively to prevent long-term consequences at time point t2. Adopted from Trinka et al. 2015 (10).

The correct choice of timing, dosage, and sequence of ASM is important, but the optimal pharmacologic treatment is largely unknown for the pediatric population and most guidelines focus on adult patients. Only a few randomized-controlled trials (RCT) exist for children that meet class I evidence (15, 16). Therefore, pharmaceutical management guidelines such as the ILAE pocket card^1^ often rely on expert opinions or experience with adult patients, giving rise to the need of controlled studies in pediatric patients and neonates.

Two studies identified that time until sufficient treatment is established is often prolonged and if treatment is started it is often not sufficiently high, partly due to out-of-hospital onset and intermittent SE (17–20). This calls for the establishment and distribution of guidelines, particularly for children, to aggressively address the treatment delay and to reduce putative permanent neuronal damage.

**Figure 1:**
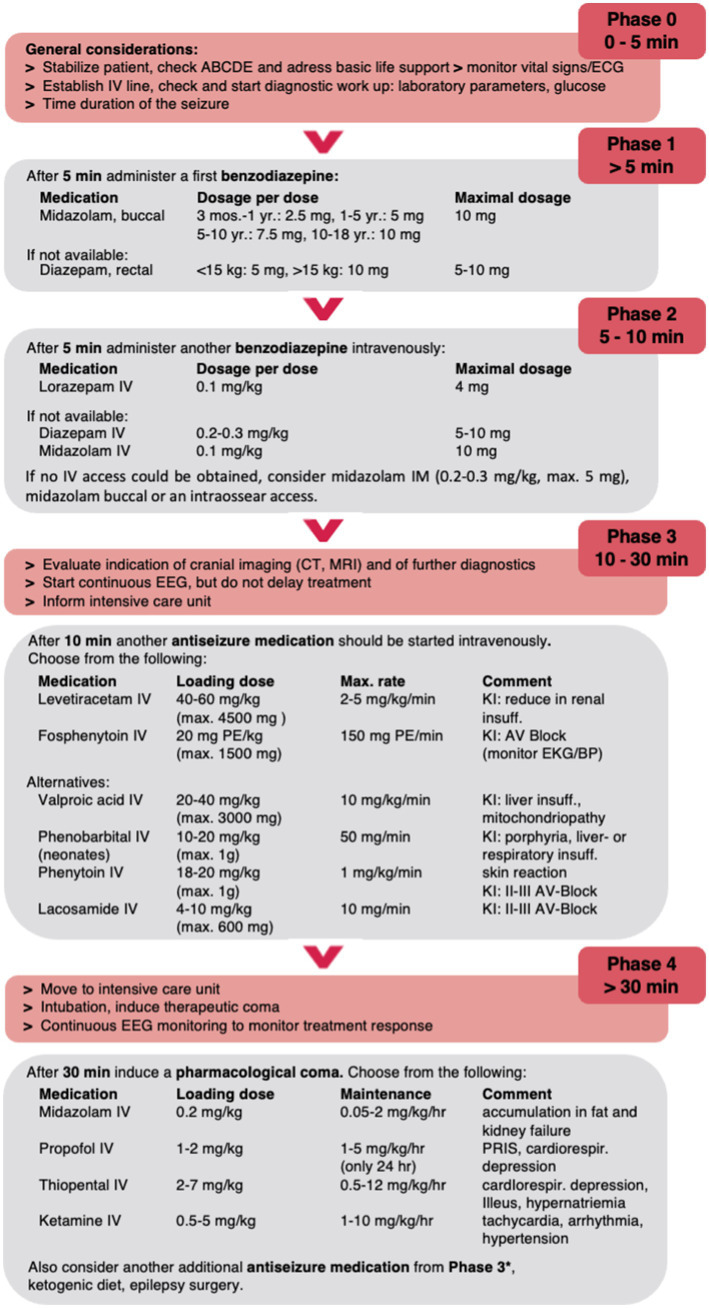
Proposed treatment algorithm for pediatric convulsive SE. Please note that this algorithm is designed to assist but not dismiss clinicians of their medical judgement of individual patient conditions and may need to be modified. The dose recommendations may vary between countries and guidelines; maximum doses are given in parentheses. *E.g., lacosamide IV or phenobarbital IV in high doses up to 140 mg/kg/d. PR, per rectum; B, buccal; IV, intravenous; IN, intranasal; IM, intramuscular; PE, phenytoin equivalent.

In the following, we summarize the current data on pharmacologic treatment of convulsive SE in childhood and propose a treatment algorithm for pediatric SE excluding neonates due to the absence of studies (Figure 1).

## Methods

A literature search was performed using PubMed (1978 until August 30th, 2022) with the MeSH Terms “status epilepticus” and “child” selecting only clinical studies, clinical trials, RCT, and observational clinical studies. Additionally, ClinicalTrails.org was searched by completed studies on “status epilepticus in children” (last August 30th, 2022). All prospective and retrospective studies including >2 children (age < 18 years) comparing two and more treatment options for status epilepticus were included. Studies were excluded if only one patient was reported, two drug treatment regimens were not compared, or the publication language was not English. Information on the number of patients, the study type (retrospective, single/multi center, randomized controlled study, etc.), and treatment regime including dosage and duration of treatment and outcome (cessation of status epilepticus) were extracted. The flow diagram in Supplementary Figure S1 shows the review process in line with the PRISMA guidelines. Additional literature was identified by evaluating available reviews and flowcharts on this topic. The literature was divided into the commonly used treatment algorithm phases and summarized (Supplementary Table S1). No statistical analysis or meta-analyses was performed and the review was not registered; no protocol was prepared.

## Results

### General considerations (phase 0, 0–5 min)

Basic life support (airway, breathing, circulation, disability) needs to be addressed in every patient with a seizure. This includes monitoring of vital parameters, heart rate, and oxygen saturation. It is essential to start timing a seizure from the very beginning to intensify treatment appropriately (12, 16).

The seizure semiology details should be recorded, and seizure type(s) determined. A detailed history about preexisting conditions/ comorbidities including an epilepsy diagnosis or recurrent status epilepticus with previous or existing ASM dosages should be conducted. An extended neurological exam should be performed as soon as possible to identify focal neurologic deficits and specific clinical signs that indicate a seizure etiology.

The initial work-up can be initiated while establishing intravenous (IV) access and should include the analysis of blood gas, electrolytes, and glucose levels, a blood count, a toxicology screen, and ASM levels, if appropriate. If IV access cannot be obtained, intraosseous (or central) access should be pursued (16).

An electroencephalogram (EEG) should be sought as early as possible. An EEG can help identify a focal seizure onset, clarify non-motor seizures or paroxysmal events (under muscle relaxing drugs), and monitor sedation (12).

Cranial imaging studies using magnetic resonance imaging (MRI) or computer tomography (CT) need to be considered in every child with SE, particularly in children without previously history of epilepsy, brain surgery, an implanted shunt systems, or in those at risk of complications that might result in SE (16, 21, 22). Imaging can reveal tumors, inflammation, ischemia, cerebrospinal fluid (CSF) retention with herniation, and other possible patterns important for treatment initiation and ultimately outcome.

### Early phase treatment in the pre-hospital setting (phase 1, >5 min)

The first-line treatment for children with convulsive SE in the pre-hospital setting is a non-IV applied benzodiazepine (Figure 1). Available benzodiazepines are diazepam (rectal, IV), midazolam (buccal, intramuscular (IM), intranasal (IN), IV), and lorazepam (IV, IN, buccal) without evidence for the best agent and application in children (23).

Rectal diazepam is usually given at a dose of 0.2–0.5 mg/kg (proposed doses <15 kg 5 mg, >15 kg 10 mg, maximum single dose 10 mg, maximum two doses). Diazepam is lipophilic and rapidly penetrates the blood–brain barrier, leading to onset within a few minutes, a maximum effect after 10–20 min, and a long half-life of 20–100 h (24). Buccal midazolam is given at 0.2–0.5 mg/kg (proposed doses: 3 months - < 1 year 2.5 mg, 1– <5 years 5 mg, 5– <10 years 7.5 mg, >10 years 10 mg, maximum single dose 10 mg, maximum two doses) with a maximum effect expected after approximately 10 min and a shorter half-life than diazepam of 3–4 h (24). In a recent Cochrane review on drug management of children with GTCS including convulsive SE, no evidence for efficacy between the latter two drugs was identified (24). Another meta-analysis on midazolam, lorazepam, and diazepam for the treatment of SE in children found a superior effect for non-IV midazolam (buccal, IN, or IM) in comparison to non-IV diazepam (23). In conclusion, buccal midazolam has been reported to be at least as effective in cessation of SE, equally safe, and also more socially acceptable than rectal diazepam (10, 15, 16, 25–33). Buccal midazolam is also the most cost-effective drug in the United States (34).

There is insufficient evidence to support the use of alternatives such as IN (or IM) midazolam and IN or buccal lorazepam to buccal midazolam or rectal diazepam in the pre-hospital setting (15). Previous studies, however, have suggested that IN midazolam (0.2 mg/kg/dose) is more effective than rectal diazepam (0.5 mg/kg/dose) (35–37). McTague et al. highlighted that in general buccal and IN application of ASMs resulted in similar seizure cessation rates as IV-applied ASMs (15, 38–44). The anticonvulsant effect of sublingual lorazepam has been reported to start with a delay of about 20 min after drug application, and this treatment is therefore inappropriate for acute SE treatment (45). Paraldehyde (IM, rectal) is not recommended anymore due to less availability in clinics and also superiority of currently accepted treatments (15, 46).

Due the efficacy of buccal midazolam, the data on cost-effectiveness of midazolam, and the ease of administration through the buccal routes, we propose to use buccal midazolam as the best option for prolonged seizures >5 min in a pre-hospital setting (Figure 1).

### Early phase treatment in the hospital setting (phase 2, 5–10 min)

In most guidelines the first drug treatment phase after stabilization of the patient in the emergency hospital setting is the administration of a benzodiazepine. The effect of benzodiazepines can decrease drastically as SE progresses due to GABA_A_ receptor internalization (14). Therefore, early treatment and sufficient dosage are essential. If benzodiazepines such as buccal midazolam or rectal diazepam have already been administered in the pre-hospital setting, care should be given not to overdose, i.e., apply more than two doses including the pre-hospital doses and thereby exceeding the maximum dosage (Figure 1) (47). An excess benzodiazepine exposure increases the risk of respiratory depression (47).

There is still limited data regarding the possible superior effect of IV versus non-IV benzodiazepine administration, especially midazolam (15, 16, 23). In general, IV lorazepam, midazolam, and diazepam result in similar rates of seizure cessation and respiratory depression, but lead in some studies to more rapid seizure cessation than buccal midazolam or rectal diazepam (48, 49). Another possible administration is IM midazolam. In the multi-center, double-blind, RCT RAMPART trial (Rapid Anticonvulsant Medication Prior to Arrival Trial), IM midazolam (13–40 kg: 5 mg) was as effective as IV lorazepam (13–40 kg: 2 mg) in SE treatment in the pre-hospital setting in a mixed adult and pediatric cohort of 893 patients including 120 children (73% versus 63%) (50–52). This result was not statistically significant when studying only the 120 children, and the prolonged time to achieve an IV line for the application of lorazepam needs to be considered. This result is supported by two further meta-analyses including the Cochrane review with similar or better seizure cessation rate of IM midazolam than IV diazepam/lorazepam (15, 23, 53–55).

If an IV access has been obtained, IV lorazepam (0.1 mg/kg/dose, maximum single dose 4 mg, maximum two doses), IV diazepam (0.2–0.3 mg/kg/dose, maximum single dose 10 mg, maximum two doses), or IV midazolam (0.1 mg/kg/dose, maximum single dose 5 mg) should be considered (24). The effect of these drugs should be apparent rapidly, within 0.5–5 min. There is insufficient evidence to favor either of these IV drugs with respect to seizure control (15, 48). In addition, there is no clear significant difference between the SE cessation effect of IVs midazolam versus IV diazepam, IV midazolam versus IV lorazepam, or IV lorazepam versus combined IV diazepam/phenytoin (15, 27, 56–58). However, a second dose to control seizures had to be applied less often if lorazepam was given compared to diazepam; no significant difference was seen when comparing the effect of midazolam versus lorazepam or diazepam versus midazolam (48). IV lorazepam has been associated with fewer adverse events, such as respiratory depression, excessive somnolence, and sedation, than IV diazepam (15, 59). Favoring lorazepam as the first-line treatment over diazepam has been criticized recently due to limited data on a superior effect (58, 60).

If the seizure does not terminate 5 min following initial benzodiazepine administration, then a second benzodiazepine dose should be administered. An application of more than two consecutive doses of benzodiazepines (including any dose given in the pre-hospital setting) increases the risk of respiratory depression and is associated with sedation (47, 61).

In conclusion, we suggest applying IV lorazepam if IV access is available given that it is at least as effective as or more effective than IV diazepam/midazolam and has been suggested to have fewer side effects. If no IV access is available, buccal and especially IM midazolam are also acceptable first-line anticonvulsants for convulsive SE treatment in the hospital setting (Figure 1).

### Expanded treatment in the hospital setting (phase 3, >10 min)

When benzodiazepines fail to terminate a convulsive SE, non-benzodiazepine ASM such as phenytoin, fosphenytoin, and phenobarbital are applied (Figure 1). There are no RCTs comparing the voltage-gated sodium channel inhibitor fosphenytoin to the positive allosteric GABA_A_ receptor modulator phenobarbital in children, but fosphenytoin is usually preferred in pediatric SE treatment guidelines (except in neonates where phenobarbital is preferred), given its fewer cardiorespiratory depression side effects when compared to phenobarbital given after benzodiazepines (16, 62). Phenobarbital can cause respiratory depression, hypotension, and bradycardia and should thus be given only in an intensive care setting. When phenobarbital and fosphenytoin are used sequentially, fosphenytoin has been suggested to precede phenobarbital, especially when benzodiazepines have already been used, on account of its better safety profile and the lower likelihood of cardiorespiratory depression (62, 63). There is insufficient data about the comparative efficacy of phenytoin and fosphenytoin; however, fosphenytoin is better tolerated compared with phenytoin with respect to cardiac arrhythmias, blood pressure imbalance, and local skin reactions (16). Phenytoin and fosphenytoin are hepatic enzyme inducers and can subsequently lower other drug levels such as those of carbamazepine, oxcarbazepine, valproate, levetiracetam, lacosamide, lamotrigine, and topiramate (64, 65). Despite fosphenytoin (and phenytoin) being contraindicated in the daily treatment of Dravet syndrome, there is currently no proven contraindication in the acute setting though mostly other ASMs are applied (66).

There is increasing evidence to support the use of alternatives to IV fosphenytoin and IV phenobarbital for treatment of pediatric convulsive SE such as the off-label administration of levetiracetam, briveracetam, valproic acid, and lacosamide. These drugs are frequently applied when registered treatment options fail. There is some evidence that there is no difference in whether these second-line ASMs are already in the patients’ home medication or not (67).

For levetiracetam, several RCTs have shown its benefit in treatment of pediatric convulsive SE despite not being approved for this application. In a recent trial, a superior effect of levetiracetam (40 mg/kg/dose) over phenytoin (20 mg/kg/dose) (93% versus 83%) in cessation of benzodiazepine-refractory SE was demonstrated in a large cohort of 600 children (mean age 3–4 years) (68). This superior effect could not be confirmed in the ConSEPT trial (Convulsive Status Epilepticus Pediatric Trial) (69). In the latter, 233 children (3 months - 13 years) who presented to one of 13 emergency departments in Australia and New Zealand over a 2.5-year time period were treated with IV or intraosseous infusions of phenytoin (20 mg/kg/dose) or levetiracetam (40 mg/kg/dose). Clinical cessation of seizure activity 5 min after the end of the drug infusion occurred in 60% of patients in the phenytoin group and 50% of patients in the levetiracetam group. It needs to be noted, however, that the infusion times varied between the two groups (20 min for phenytoin, 5 min for levetiracetam). Given that SE duration comes with more difficulties in controlling SE this can be a bias. In the EcLiPSE trial (Emergency Treatment with Levetiracetam of Phenytoin in Status Epilepticus), 404 children (6 months −18 years) who presented with SE to one of 30 emergency departments in the UK over a 3-year time period were treated similarly to the ConSEPT with phenytoin (20 mg/kg/dose) or levetiracetam (40 mg/kg/dose) through IV or intraosseous infusions with differing infusion times (70). Deviating from the ConSEPT trial, the primary outcome was not seizure cessation 5 min after end of the drug infusion, but rather time from randomization to cessation of convulsive SE. Here, convulsive SE occurred in 64% of patients in the phenytoin group within a median time of 45 min and 70% of patients in the levetiracetam group within a median time of 35 min. The authors conclude that though no significant superiority of levetiracetam over phenytoin was identified, levetiracetam could be an appropriate alternative to phenytoin as the first-choice, second-line ASM in the treatment of pediatric convulsive SE given their data and the safety profiles of the drugs (70). In the most recent large double-blind RCT ESETT (Established Status Epilepticus Treatment Trial), the efficacy of fosphenytoin (20 mg/kg, max. 1,200 mg), valproate (40 mg/kg, max. 3,000 mg), and levetiracetam (60 mg/kg, max. 4,500 mg) in 225 children >2 years and 237 adults with benzodiazepine-refractory convulsive SE were included. There was no difference in efficacy or safety in children between the three groups with seizure ceasing within 1 h with levetirazetam in 52%, with fosphenytoin in 49%, and with valproate in 52% of patients (71–73). Other studies also underlined the similar effect of levetirazetam in comparison to valproate and fosphenytoin, and it being well tolerated in general (74–76).

In conclusion, levetiracetam is overall well tolerated with only low rates of increased aggressiveness, irritability, nausea, and vomiting. In our own hospital setting, off-label levetiracetam is an equally established drug to fosphenytoin in children given the low side-effect profile.

In adults, brivaracetam has been reported to be a safe alternative to levetiracetam to treat SE (77, 78), but results of an ongoing RCT for children are not available yet, except for one study that includes pediatric cases with absence SE (79). Given the faster transition of the brain–blood barrier of brivaracetam versus levetiracetam, reaching maximum concentration in the brain within minutes following IV application, brivaracetam treatment is a promising approach, however, it is currently not approved for SE treatment (80).

Sodium valproate, which modulates sodium and calcium channels and the metabolism of GABA, has been shown to be effective in treatment of pediatric convulsive SE and in adults to be superior in SE cessation (e.g., than phenytoin) (10, 16, 64, 81, 82). In a RCT on 100 children with diazepam-refractory SE, valproic acid (20 mg/kg/dose) and phenytoin (20 mg/kg/dose) were similarly efficient in ceasing seizure activity with no significant difference in side-effects (83). In a further RCT, IV valproic acid (20 mg/kg/dose) was more effective in SE cessation (90% versus 77% seizure cessation) and better tolerated (adverse effects 24% versus 74%) than IV phenobarbital (20 mg/kg/dose) in 60 children younger than 2 years-of-age (84). Sodium valproate can be administered rapidly IV through an infusion pump with rare adverse effects such as hypotension, blood count drop, platelet dysfunction, hypersensitivity, (acute hemorrhagic) pancreatitis, and hyperammonemia (85). A major concern is valproic acid hepatotoxicity, particularly in children who are under 2 years of age and who could have a metabolic/mitochondrial disorder. Particularly, mutations in the *DNA polymerase gamma gene* (*POLG*) cannot be ruled out in the acute setting unless in-depth genetic testing has already been performed and results are readily available (86). Valproate is a strong hepatic enzyme inhibitor, and it may raise other drug levels such as those of carbamazepine, lamotrigine, phenobarbital, and rufinamide (85, 87). Especially when combining with drugs favoring hyperammonemia, e.g., phenobarbital, clinicians should be concerned about valproate-induced hyperammonemic encephalopathy, a rare complication characterized by decreased consciousness, neurological deficits, and vomiting (88).

Only a few studies focused on the effect of lacosamide in the treatment of pediatric SE, most likely due to the fact that lacosamide is licensed for children older than 4 years and focal-onset seizures only (not for SE treatment) and the subsequent off-label use in younger children (89). A review on six retrospective studies including 36 pediatric patients with various SE subtypes showed an overall success rate of 45–78% (age range 1 month to 17 years) with doses between 4 to 10 mg/kg/dose (89). Of the 36 lacosamide-treated children, one had bradycardia and two had delayed oculogyric crisis and chorea. Otherwise, no serious side effects were reported. Since lacosamide can cause PR/QT prolongation, it should be administered with electrocardiogram (ECG) monitoring (89, 90). Lang et al. recently reported a 70% cessation rate of seizure series or SE in a heterogenous mixed pediatric and adult cohort of 119 patients who received additional lacosamide (median dose 300 mg) to the hospital’s ASM protocol with a good tolerability (91). In a study with 196 adult and pediatric patients, lacosamide and levetirazetam had a higher SE cessation rate than valproate and phenytoin as a first or second ASM in benzodiazepine-refractory SE (92). Overall, due to missing RCT studies comparing lacosamide in children to approved ASMs, no recommendation can be made at this point. In our hospital, lacosamide is applied as an ASM in phase 4.

In conclusion, we suggest applying IV levetiracetam or IV fosphenytoin; alternatively, valpoic acid IV or phenobarbital can be used (Figure 1).

### Treatment of refractory pediatric SE in the hospital intensive care setting (phase 4, >30 min)

In approximately 10–40% of children, the first- and second-line drugs fail to stop convulsive SE, and the SE is regarded as refractory (RSE). In 7% of children, the SE develops into super-refractory SE (SRSE), where the SE is ongoing after 24 h into induction of a pharmacological coma or proceeding after its withdrawal (11). Both events are highly associated with long-term neurological morbidity and mortality (RSE: 16–43.5%). Therefore, aggressive treatment of SE at this time point (t2) is essential to avoid permanent neuronal damage. There is no clear evidence to guide pharmaceutical treatment in this phase. Therefore, treatment is based on case series and expert opinions without controlled trials being available (11). Additionally, specific treatment for the underlining etiology should be started (see chapter: pharmacological considerations in specific pediatric SE subtypes).

Most patients are transferred at this point to the intensive care unit to receive further second-line drugs and/or general anesthetic drugs such as thiopental, pentobarbital, midazolam, or propofol (third-line agents) with continuous EEG (cEEG) monitoring (16, 93, 94) (Figure 1). There are limited data supporting which third-line ASM to choose and the recommended speed of titration (11). cEEG is essential to guide the pharmacological coma as well as exclusion of non-convulsive SE (NCSE) or electrographic seizures. It is unclear whether the treatment goal should merely be the termination of seizures or the induction of a burst-suppression pattern. In addition, it remains unclear how long a patient should be maintained in a pharmacologic coma. Expert opinion usually opts for 24–48 h of electrographic seizure control prior to a gradual withdrawal of continuous infusions (11, 95–97). If seizures continue despite treatment or recur during the weaning period of continuous infusion(s), a further trial for an additional 24–48 h is usually recommended (11).

In a midazolam-induced coma, dosing usually involves an initial loading dose of 0.2 mg/kg in a 2 mg/min infusion followed by an infusion at 0.05–2 mg/kg/h titrated as needed to achieve clinical and electrographic seizure suppression and/or EEG burst-suppression (11, 98). Pentobarbital is usually initially given at a loading dose of 5 mg/kg in a 50 mg/min infusion, and a further pulse of 5 mg/kg at similar 50 mg/min infusion speed can be subsequently administered, if needed (10, 11) This is followed by an infusion at 0.5–5 mg/kg/h that is titrated as needed to achieve clinical and electrographic seizure suppression and/or EEG burst-suppression (10, 11, 99, 100). If seizures persist with midazolam or pentobarbital, then escalating dosing through additional boluses is needed to rapidly increase levels or terminate seizures (98). Propofol (up to 5 mg/kg/h) is an additional safe and effective option, but recommended for use only for 24 h since it may cause propofol infusion syndrome (PRIS), which is associated with a high mortality rate (101, 102). Another possibility is the usage of inhalational anesthetics such as isoflurane to terminate an SRSE. While two pediatric clinical series reported seizure cessation in 94.4%, there is, unfortunately, a high relapse rate after discontinuing the treatment (103).

Though no RCT trials exist, very high-dose phenobarbital has been reported by several authors as an approach to control RSE. In our hospital, we increase the dose of phenobarbital to escalate plasma levels of 80 ug/ml. This is in line with previous reports where dosages ranged from 40 to 140 mg/kg/d with plasma levels of 30–340 ug/ml (104–107).

A promising future therapeutic approach in RSE is treatment with the noncompetitive NMDA receptor antagonist ketamine or (S)-ketamine (loading: 0.5–5 mg/kg, maintenance 1–10 mg/kg/h) (108). Only a small number of case series exist for such treatment in children (108). In adults, data on (S)-ketamine are available from open observational studies with response rates of up to 64% (108). A prospective study (KETASER01) comparing the effectiveness of ketamine to diazepam, thiopental, and propofol in children (1–18 years) in RSE is currently being conducted (109, 110).

Additional ASM application (e.g., phenytoin, valproate, levetiracetam, or topiramate) can be reasonable if these drugs have not been tried, if seizures become less frequent, or if they appear to be fragmenting (111). Further options including cannabidiol, bexanolone, ketogenic diet, and hypothermia cannot be classified as standard therapy but may become options when other measures fail to cease SE (93–95, 112–117). In any case of RSE due to structural epilepsy, epilepsy surgery needs to be considered urgently.

### Pharmacological considerations in specific pediatric SE subtypes

Specific pediatric SE subtypes require specific considerations with respect to pharmacologic treatment. In tonic SE, benzodiazepines should be avoided and therapy may be initiated with fosphenytoin or phenobarbital (or levetiracetam) treatment. Absence SE can often be stopped through treatment with clonazepam, phenobarbital, or valproate. In febrile prolonged seizures, the identification of the primary fever source is essential. A lumbar puncture can help diagnose meningoencephalitis and help in decision making with respect to early antiviral or antibacterial treatment (11).

Early genetic consultation and, if available, rapid whole exome/genome sequencing should be initiated if a genetic etiology is suspected to offer individualized treatment options (118).

In patients with unknown or autoimmune etiologies and non-infectious encephalitis, extensive diagnostic work-up should be performed. Treatment with IV immunoglobulins (1–2 g/kg) over 3–5 days should be considered after securing sufficient blood and CSF sample specimens for later analysis. As soon as infectious encephalitis has been ruled out, IV corticosteroids (methylprednisolone: 20–30 mg/kg/d for 3–5 days) and plasmapheresis or immunoabsorption need to be considered (11).

Seizures in febrile infection-related epilepsy syndrome (FIRES) are highly difficult to treat and usually remain refractory to standard therapy (116, 119). Treatment approaches include ASM, ketogenic diet, and pharmacologic coma induction. Given the putative causal role of inflammation in FIRES, immunomodulatory therapies have been initiated such as IV corticosteroids, IV immunoglobulins, plasma exchange or immunoabsorption, and/or further drug treatment with, e.g., tacrolimus, rituximab, cyclophosphamide, and anakinra (116, 119).

## Conclusion

In SE the correct choice of timing, dosage, and sequence of ASM is important, but the optimal pharmacologic treatment is largely unknown for the pediatric and especially neonatal population as most guidelines focus on adult patients. Although it is known that prolonged seizures are more difficult to treat the longer they continue, as this can lead to permanent brain damage and death, sufficient treatment is often delayed and if treatment is started it if often not sufficiently high.

Limitations of this study are that the review does not include meta-analyses and statistics and includes the recommendation from pre-existing systematic reviews. The study is also limited by the number of identified studies.

This study and our proposed algorithm therefore highlight the immense importance of the establishment and distribution of guidelines, particularly for children, to aggressively address the treatment delay and to reduce putative permanent neuronal damage.

## Author contributions

AK and LLB drafted the initial manuscript and performed the systematic literature search, which was reviewed and revised by AG, CP, and CE. All authors approved the final manuscript as submitted and agree to be accountable for all aspects of the work.

## Funding

Our research is supported by the German Research Foundation (DFG; SFB1315, FOR3004) and the Günter Endres Fond through the Einstein Foundation.

## Conflict of interest

The authors declare that the research was conducted in the absence of any commercial or financial relationships that could be construed as a potential conflict of interest.

## Publisher’s note

All claims expressed in this article are solely those of the authors and do not necessarily represent those of their affiliated organizations, or those of the publisher, the editors and the reviewers. Any product that may be evaluated in this article, or claim that may be made by its manufacturer, is not guaranteed or endorsed by the publisher.

## Footnotes

^1^ https://www.ilae.org/files/dmfile/StatusEpilepticus_pocket_card.pdf
