# Supplementary material for: Treatment of pediatric convulsive status epilepticus
Source: Front Neurol. 2023 Jun 29;14:1175370. doi: 10.3389/fneur.2023.1175370 (PMC10343462; doi:10.3389/fneur.2023.1175370)
Supplement: Supplementary file 1 [file Data_Sheet_1.pdf]

Supplementary Material

Treatment of Pediatric Convulsive Status Epilepticus

Lena-Luise Becker,[1-3], MD, Alexander Gratopp,[4], MD, Christine Prager,[1-2], MD, Prof. Christian E. Elger,[1-2,5], MD, Prof. Angela M. Kaindl[1-3*], PhD

***Correspondence**: Prof. Dr. Angela M. Kaindl: angela.kaindl@charite.de

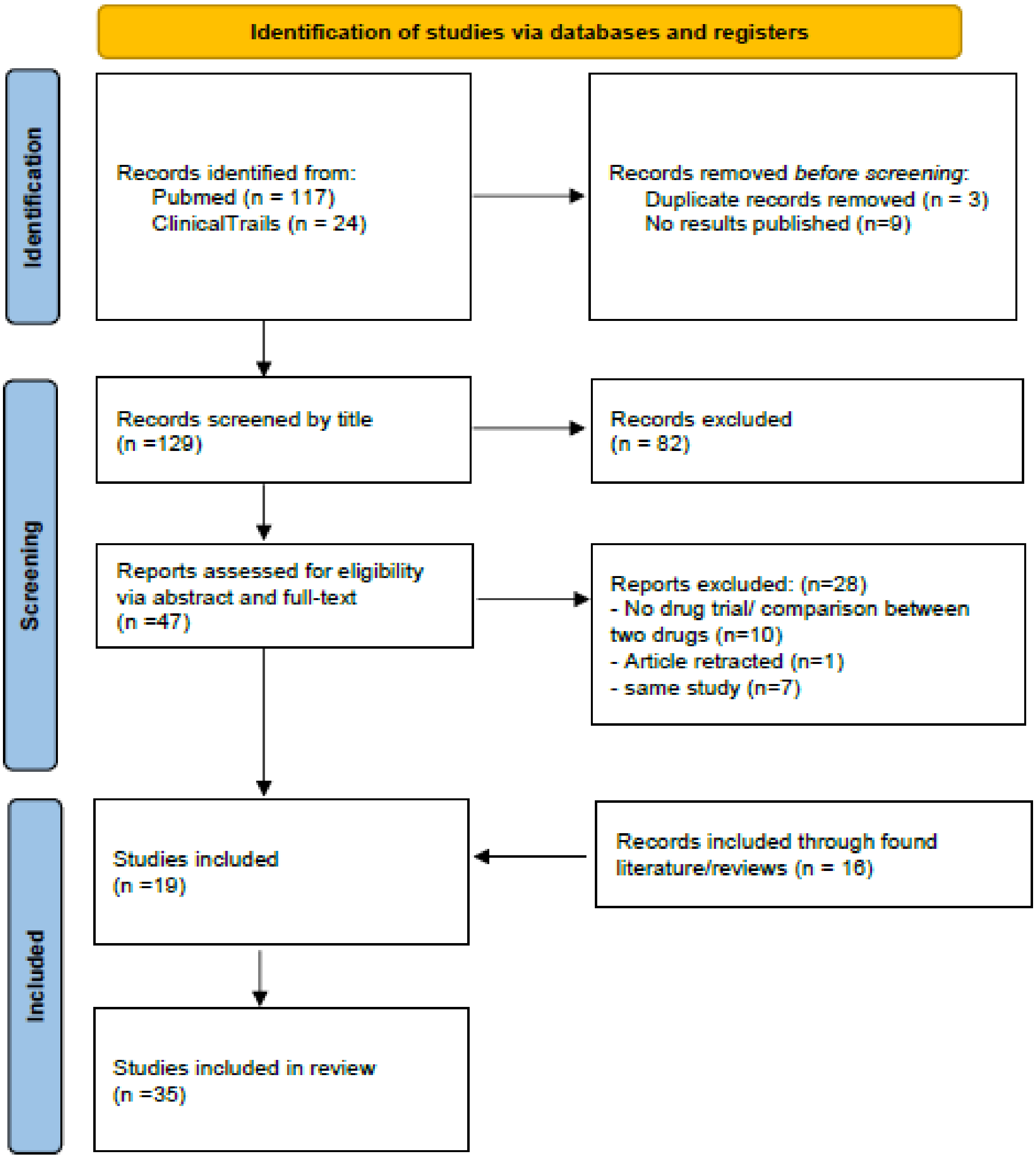

Supplemental Figure 1. PRISMA flowchart. *From:* Page MJ, McKenzie JE, Bossuyt PM, Boutron I, Hoffmann TC, Mulrow CD, et al. The PRISMA 2020 statement: an updated guideline for reporting systematic reviews. BMJ 2021;372:n71. doi: 10.1136/bmj.n71.

Study	Study type	No. of pat. or doses*	Age range	Status type	Treatment			Outcome:
					regime	admin.	dosage	Seizure cessation (within 10 min*) (N)
**Phase 1/2**								
Mpimbaza, Ndeezi et al. 2008(1)	Single-blind RCT	165<br>165	3 m-12 yr	SE	midazolam<br>diazepam	buccal<br>rectal	Typical dose	115*<br>93*
McIntyre, Robertson et al. 2005(2)	Multicenter RCT	109*<br>110*	6 m-15 yr.	SE	midazolam<br>diazepam	buccal<br>rectal	Typical dose	61*<br>30*
Momen, Azizi Malamiri et al. 2015(3)	RCT	50<br>50	1 m-16 yr	SE	midazolam<br>diazepam	IM<br>rectal	0,3 mg/kg<br>0,5 mg/kg	48<br>47
Scott, Besag et al. 1999(4)	RCT	40*<br>39*	5 yr-22yr	SE	midazolam<br>diazepam	buccal<br>rectal	10mg<br>10mg	30<br>23
Ashrafi, Khosroshahi et al. 2010(5)	RCT	49<br>49	3 m-12 yr	SE	midazolam<br>diazepam	buccal<br>rectal	Typical dose	49*<br>40*
Fisgin, Gurer et al. 2002(6)	Quasi-RCT	22<br>23	1 m-13 yr	SE	diazepam<br>midazolam	Rectal<br>IN	0,3 mg/kg<br>0,2 mg/kg	13*<br>20*
Baysun, Aydin et al. 2005(7)	Quasi-RCT	23<br>23	2 m-12 yr	SE	midazolam<br>diazepam	buccal<br>rectal	0.25 mg/kg<br>0.3-0.5 mg/kg	18*<br>17*
Rudra, Ghosh et al. 2021(8)	Observational study	80<br>80	5-12 yr	SE	lorazepam	IN<br>IV	0.1 mg/kg<br>0.1 mg/kg	60<br>66
Arya, Gulati et al. 2011(9)	RCT	70<br>71	6-14 yr	SE	lorazepam	IN<br>IV	0.1 mg/kg<br>0.1 mg/kg	59<br>56
Lahat, Goldman et al. 2000(10)	RCT	26<br>26	6 m-5 yr	Febrile SE	midazolam<br>diazepam	IN<br>IV	0.2 mg/kg<br>0.3 mg/kg	23<br>24
Mahmoudian and Zadeh 2004(11)	RCT	35<br>35	3 m-12 yr	SE	midazolam<br>diazepam	IN<br>IV	0.2 mg/kg<br>0.2 mg/kg	35<br>35
Javadzadeh, Sheibani et al. 2012(12)	RCT	30<br>30	2 m-15 yr	SE	midazolam<br>diazepam	IN<br>IV	0.2 mg/kg<br>0.3 mg/kg	30<br>30

Talukdar and Chakrabarty 2009(13)	RCT	60<br>60	0-12 yr	SE	midazolam<br>diazepam	buccal<br>IV	0.2 mg/kg<br>0.3 mg/kg	51<br>56
Ahmad, Ellis et al. 2006(14)	RCT	80<br>80	2 m-12 yr	SE	lorazepam<br>paraldehyde	IN<br>IM	0.1 mg/kg<br>0.2 ml/kg	60<br>49
Appleton, Sweeney et al. 1995(15)	Quasi-RCT	27/6<br>34/19	< 16 yr	SE	lorazepam<br>diazepam	IV/rectal<br>IV/rectal	0.05-1 mg/kg<br>0.3-0.4 mg/kg	19/6<br>22/6
Chamberlain, Okada et al. 201(16)	RCT, double blind	140<br>133	3 m-18 yr	SE	diazepam<br>lorazepam	IV<br>IV	0.2 mg/kg<br>0.1 mg/kg	101<br>97
Gathwala, Goel et al. 2012(17)	RCT	40<br>40<br>40	6 m-14 yr	SE	diazepam<br>midazolam<br>lorazepam	IV<br>IV<br>IV	0.3 mg/kg<br>0.1 mg/kg<br>0.1 mg/kg	29<br>36<br>38
Chamberlain, Altieri et al. 1997(18)	RCT	13<br>11	0 m-18 yr	SE	midazolam<br>diazepam	IM<br>IV	0.2 mg/kg<br>0.3 mg/kg	12<br>10
Welch, Nicholas et al. 2015(19)	RCT	60<br>60	< 18 yr	SE	midazolam<br>lorazepam	IM<br>IV	RAMPART protocol	41<br>43
Sreenath, Gupta et al. 2010(20)	RCT	90<br>88	1-12 yr	SE	lorazepam<br>diazepam<br>phenytoin	IV/rectal<br>IV/rectal<br>IV	0.1 mg/kg<br>0.2 mg/kg<br>18 mg/kg	6<br>14
Shah and Deshmukh 2005(21)	Partly-RCT	50<br>65	1 m-12 yr	SE	midazolam<br>diazepam	IM<br>IV	0.3 mg/kg<br>0.2 mg/kg	45<br>63
Giang, McBride, 1988 (22)	Retrospective	16<br>22	2 w-18 yr	SE > 20 min	lorazepam<br>diazepam	IV<br>IV	mean: 0.11 mg/kg<br>mean: 0.38 mg/kg	11<br>18
Chin, Neville et al, 2008(23)	Prospective clinical trial	107*<br>80*	29 d-15 yr	SE	lorazepam<br>diazepam	IV<br>rectal	0.1 mg/kg<br>0.5 mg/kg	63<br>56

**Phase 3- BZR-SE**								**Seizure cessation (including within infusion time to 30-60 min after infusion stop**)
Noureen, Khan et al. 2019(24)	RCT	300<br>300	<18 yr	BZR-SE	levetiracetam<br>phenytoin	IV<br>IV	40 mg/kg over 15 min<br>20 mg/kg over 30 min	278<br>250
Chamberlain, Kapur et al. 2020(25)	RCT, double-blind (ESETT)	85<br>71<br>69	2 yr-18 yr	BZR-SE	levetiracetam<br>fosphenytoin<br>valproate	IV<br>IV<br>IV	60 mg/kg over 10 min<br>20 mg/kg over 10 min<br>40 mg/kg over 10 min	44<br>35<br>36
Lyttle, Rainford et al. 2019(26)	RCT (EcLiPSE)	152<br>134	6 m-18 yr	BZR-SE	levetiracetam<br>phenytoin	IV<br>IV	40 mg/kg over 5 min<br>20 mg/kg over 20 min	106<br>86
Dalziel, Borland et al. 2019(27)	RCT (ConSEPT)	114<br>119	3 m-16 yr	BZR-SE	phenytoin<br>levetiracetam	IV/IO<br>IV/IO	20 mg/kg over 20 min<br>40 mg/kg over 20 min	68<br>60
Agarwal, Kumar et al. 2007(28)	RCT	50<br>50	> 2 yr (also adult)	BZR-SE	phenytoin<br>valproate	IV<br>IV	20 mg/kg -40 mg/min<br>20 mg/kg -50 mg/min	44<br>42
Vignesh, Rameshkumar et al. 2020(29)	RCT, double-blind	35<br>35<br>32	3 m-12 yr	BZR-SE	phenytoin<br>valproate<br>levetiracetam	IV<br>IV<br>IV	20 mg/kg over 20 min<br>20 mg/kg over 20 min<br>20 mg/kg over 20 min	31<br>29<br>30
Misra, Dubey et al. 2017(30)	RCT	35<br>33	Children and adult	BZR-SE	phenytoin<br>valproate	IV<br>IV	18 mg/kg- 50 mg/min<br>30 mg/kg over 15 min	23<br>14
Nalisetty, Kandasamy et al. 2020(31)	RCT	29<br>32	2 m-18 yr	BZR-SE	levetiracetam<br>fosphenytoin	IV<br>IV	40 mg/kg over 10 min<br>20 mg/kg over 10 min	30<br>20
Malamiri, Ghaempanah et al. 2012(32)	RCT	30<br>30	2-18 yr	BZR-SE	valproate<br>phenobarbital	IV<br>IV	20 mg/kg –60- 100 mg/min<br>20 mg/kg – 5-6 mg/min	27<br>23
Chin, Neville et al, 2008(23)	Retrospective	42*<br>32*<br>5*	29 d-15 yr	BZR-SE	paraldehyde<br>phenytoin<br>phenobarbitone	IV<br>IV<br>IV	Mean: 0.28 ml/kg<br>Mean: 12.4 mg/kg<br>15-20 mg/kg	40<br>0<br>0

**Phase 4- BZR-SE and second-line refractory SE**								**Seizure Cessation**
Abbaskhanian, Sheidaee et al. 2021(33)	RCT	35<br>35	< 18 yr	SRSE, also non-con.	valproate<br><br>midazolam	cIV<br><br>cIV	30 mg/kg > 10 mg/kg with cIV 5 mg/kg/hr<br>10 µg/kg/min with increase every 5 min until cessation	32<br><br>30
Mehta, Singhi et al. 2007(34)	RCT	20<br><br>20	5 m -12 yr	SRSE	valproate<br><br>diazepam	IV→ cIV<br><br>cIV	30 mg/kg > 10 mg/kg with cIV 5 mg/kg/hr<br>10 µg/kg/min with increase every 5 min until cessation	16<br><br>17
Van Gestel, Blussé van Oud-Alblas et al, 2005(35)	Retrospective	22<br><br>20	< 18 yr	SRSE > 60 min	propofol<br><br>thiopental	IV<br><br>IV	1-2 mg/kg > cIV 1-5 mg/kg/hr<br>cIV (aim blood level 20 mg/ml)	14<br><br>11

Supplemental Table 1: Included studies in this review. RCT: randomized controlled trial, BZR-SE: benzodiazepine-refractory status epilepticus; SE: status epilepticus; RSE: refractory status epilepticus; SRSE: super refractory status epilepticus; IV: intravenous; cIV: continuous IV; IM: intramuscular, IN: intranasal; IO: intraosseous.Typical dose: ca. 0.5 mg/kg (2.5 mg for 3–11 months of age; 5 mg for ages 1– 4 years; 7.5 mg for ages 5–9 years; and 10 mg for ages > 10 years). RAMPART protocol: midazolam IM: <40 kg: 5 mg, > 40 kg 10 mg; lorazepam IV: <40 kg: 2 mg, > 40 kg 4 mg.

Drug	Administration	Dosage	Reference
**First-line ASM: benzodiazepine**			
diazepam	rectal<br><br>IV	0.3-0.5 mg/kg: 3-11 m: 2.5 mg; 1– 4 yr: 5 mg; 5–9 yr: 7.5 mg; > 10 yr: 10 mg<br>0.2-0.3 mg/kg	(1-3, 5-7, 23)<br><br>(10-13, 15-18, 20-22)
midazolam	buccal<br><br>IM<br>IN<br>IV	0.2-0.5 mg/kg: 3-11 m:2.5 mg; 1– 4 yr: 5 mg; 5–9 yr: 7.5 mg; > 10 yr: 10 mg<br>0.2-0.3 mg/kg<br>0.2 mg/kg<br>0.1 mg/kg	(1, 2, 5, 7, 13)<br><br>(3, 18, 19, 21)<br>(6, 10-12)<br>(17, 20)
lorazepam	IN<br>IV	0.1 mg/kg<br>0.1 mg/kg	(8, 9, 14)<br>(8, 9, 15-17, 19, 20, 22, 23)
**Second-line ASM**			
levetiracetam	IV	20-60 mg/kg over 5-20 min	(24-27, 29, 31)
phenytoin	IV	18-20 mg/kg over 20-30 min	(24, 26-28, 36)
fosphenytoin	IV	20 mg/kg over 10 min	(25, 31)
valproate	IV	20-40 mg/kg over 10-20 min	(25, 28, 29, 32, 36)
phenobarbital	IV	20 mg/kg	(32)
lacosamide	IV	4-10 mg/kg	(37)
**Third-line ASM**			
midazolam	IV	loading 0.2 mg/kg >cIV: 0.05-2 mg/kg/hr	(38)
diazepam	IV	10 µg/kg/min with increase every 5 min until cessation	(34)
propofol	IV	loading 1-2 mg/kg > cIV 1-5 mg/kg/hr	(35)
thiopental	IV	loading: 2-7 mg/kg > cIV 0.5-12 mg/kg/hr	(35, 39)
pentobarbital	IV	loading: 5-15 mg/kg >cIV 0.5-5 mg/kg/hr	(38, 40)
ketamine	IV	loading: 0.5-5 mg/kg > cIV: 1-10 mg/kg/hr	(41-43)
phenobarbital	IV	40 -140 mg/kg/d	(44-46)
valproate	IV	loading 30 mg/kg > 10 mg/kg with cIV 5 mg/kg/hr	(34)

Supplemental Table 2: Current evidence and recommendations for ASM in status epilepticus. ; IV: intravenous; cIV: continuous IV; IM: intramuscular, IN: intranasal.

References:

1. Mpimbaza A, Ndeezi G, Staedke S, Rosenthal PJ, Byarugaba J. Comparison of buccal midazolam with rectal diazepam in the treatment of prolonged seizures in Ugandan children: a randomized clinical trial. Pediatrics. 2008;121(1):e58-64.
2. McIntyre J, Robertson S, Norris E, Appleton R, Whitehouse WP, Phillips B, et al. Safety and efficacy of buccal midazolam versus rectal diazepam for emergency treatment of seizures in children: a randomised controlled trial. Lancet. 2005;366(9481):205-10.
3. Momen AA, Azizi Malamiri R, Nikkhah A, Jafari M, Fayezi A, Riahi K, et al. Efficacy and safety of intramuscular midazolam versus rectal diazepam in controlling status epilepticus in children. Eur J Paediatr Neurol. 2015;19(2):149-54.
4. Scott RC, Besag FM, Neville BG. Buccal midazolam and rectal diazepam for treatment of prolonged seizures in childhood and adolescence: a randomised trial. Lancet. 1999;353(9153):623-6.
5. Ashrafi MR, Khosroshahi N, Karimi P, Malamiri RA, Bavarian B, Zarch AV, et al. Efficacy and usability of buccal midazolam in controlling acute prolonged convulsive seizures in children. Eur J Paediatr Neurol. 2010;14(5):434-8.

6. Fisgin T, Gurer Y, Tezic T, Senbil N, Zorlu P, Okuyaz C, et al. Effects of intranasal midazolam and rectal diazepam on acute convulsions in children: prospective randomized study. J Child Neurol. 2002;17(2):123-6.
7. Baysun S, Aydin OF, Atmaca E, Gurer YK. A comparison of buccal midazolam and rectal diazepam for the acute treatment of seizures. Clin Pediatr (Phila). 2005;44(9):771-6.
8. Rudra N, Ghosh T, Roy UK. A Comparative Study on Intranasal Versus Intravenous Lorazepam in the Management of Acute Seizure in Children. Folia Med (Plovdiv). 2021;63(6):958-64.
9. Arya R, Gulati S, Kabra M, Sahu JK, Kalra V. Intranasal versus intravenous lorazepam for control of acute seizures in children: a randomized open-label study. Epilepsia. 2011;52(4):788-93.
10. Lahat E, Goldman M, Barr J, Bistritzer T, Berkovitch M. Comparison of intranasal midazolam with intravenous diazepam for treating febrile seizures in children: prospective randomised study. BMJ. 2000;321(7253):83-6.
11. Mahmoudian T, Zadeh MM. Comparison of intranasal midazolam with intravenous diazepam for treating acute seizures in children. Epilepsy Behav. 2004;5(2):253-5.
12. Javadzadeh M, Sheibani K, Hashemieh M, Saneifard H. Intranasal midazolam compared with intravenous diazepam in patients suffering from acute seizure: a randomized clinical trial. Iran J Pediatr. 2012;22(1):1-8.
13. Talukdar B, Chakrabarty B. Efficacy of buccal midazolam compared to intravenous diazepam in controlling convulsions in children: a randomized controlled trial. Brain Dev. 2009;31(10):744-9.
14. Ahmad S, Ellis JC, Kamwendo H, Molyneux E. Efficacy and safety of intranasal lorazepam versus intramuscular paraldehyde for protracted convulsions in children: an open randomised trial. Lancet. 2006;367(9522):1591-7.
15. Appleton R, Sweeney A, Choonara I, Robson J, Molyneux E. Lorazepam versus diazepam in the acute treatment of epileptic seizures and status epilepticus. Dev Med Child Neurol. 1995;37(8):682-8.
16. Chamberlain JM, Okada P, Holsti M, Mahajan P, Brown KM, Vance C, et al. Lorazepam vs diazepam for pediatric status epilepticus: a randomized clinical trial. JAMA. 2014;311(16):1652-60.
17. Gathwala G, Goel M, Singh J, Mittal K. Intravenous diazepam, midazolam and lorazepam in acute seizure control. Indian J Pediatr. 2012;79(3):327-32.
18. Chamberlain JM, Altieri MA, Futterman C, Young GM, Ochsenschlager DW, Waisman Y. A prospective, randomized study comparing intramuscular midazolam with intravenous diazepam for the treatment of seizures in children. Pediatr Emerg Care. 1997;13(2):92-4.
19. Welch RD, Nicholas K, Durkalski-Mauldin VL, Lowenstein DH, Conwit R, Mahajan PV, et al. Intramuscular midazolam versus intravenous lorazepam for the prehospital treatment of status epilepticus in the pediatric population. Epilepsia. 2015;56(2):254-62.
20. Sreenath TG, Gupta P, Sharma KK, Krishnamurthy S. Lorazepam versus diazepam-phenytoin combination in the treatment of convulsive status epilepticus in children: a randomized controlled trial. Eur J Paediatr Neurol. 2010;14(2):162-8.
21. Shah I, Deshmukh CT. Intramuscular midazolam vs intravenous diazepam for acute seizures. Indian J Pediatr. 2005;72(8):667-70.
22. Giang DW, McBride MC. Lorazepam versus diazepam for the treatment of status epilepticus. Pediatr Neurol. 1988;4(6):358-61.
23. Chin RF, Neville BG, Peckham C, Wade A, Bedford H, Scott RC. Treatment of community-onset, childhood convulsive status epilepticus: a prospective, population-based study. Lancet Neurol. 2008;7(8):696-703.
24. Noureen N, Khan S, Khursheed A, Iqbal I, Maryam M, Sharib SM, et al. Clinical Efficacy and Safety of Injectable Levetiracetam Versus Phenytoin as Second-Line Therapy in the Management of Generalized Convulsive Status Epilepticus in Children: An Open-Label Randomized Controlled Trial. J Clin Neurol. 2019;15(4):468-72.
25. Chamberlain JM, Kapur J, Shinnar S, Elm J, Holsti M, Babcock L, et al. Efficacy of levetiracetam, fosphenytoin, and valproate for established status epilepticus by age group (ESETT): a double-blind, responsive-adaptive, randomised controlled trial. Lancet. 2020;395(10231):1217-24.
26. Lyttle MD, Rainford NEA, Gamble C, Messahel S, Humphreys A, Hickey H, et al. Levetiracetam versus phenytoin for second-line treatment of paediatric convulsive status epilepticus (EcLiPSE): a multicentre, open-label, randomised trial. Lancet. 2019;393(10186):2125-34.

27. Dalziel SR, Borland ML, Furyk J, Bonisch M, Neutze J, Donath S, et al. Levetiracetam versus phenytoin for second-line treatment of convulsive status epilepticus in children (ConSEPT): an open-label, multicentre, randomised controlled trial. Lancet. 2019;393(10186):2135-45.
28. Agarwal P, Kumar N, Chandra R, Gupta G, Antony AR, Garg N. Randomized study of intravenous valproate and phenytoin in status epilepticus. Seizure. 2007;16(6):527-32.
29. Vignesh V, Rameshkumar R, Mahadevan S. Comparison of Phenytoin, Valproate and Levetiracetam in Pediatric Convulsive Status Epilepticus: A Randomized Double-blind Controlled Clinical Trial. Indian pediatrics. 2020;57(3):222-7.
30. Misra UK, Dubey D, Kalita J. A randomized controlled trial of lacosamide versus sodium valproate in status epilepticus. Epilepsia. 2017.
31. Nalisetty S, Kandasamy S, Sridharan B, Vijayakumar V, Sangaralingam T, Krishnamoorthi N. Clinical Effectiveness of Levetiracetam Compared to Fosphenytoin in the Treatment of Benzodiazepine Refractory Convulsive Status Epilepticus. Indian J Pediatr. 2020;87(7):512-9.
32. Malamiri RA, Ghaempanah M, Khosroshahi N, Nikkhah A, Bavarian B, Ashrafi MR. Efficacy and safety of intravenous sodium valproate versus phenobarbital in controlling convulsive status epilepticus and acute prolonged convulsive seizures in children: a randomised trial. Eur J Paediatr Neurol. 2012;16(5):536-41.
33. Abbaskhanian A, Sheidaee K, Charati JY. Comparison of the effect of continuous intravenous infusion of sodium valproate and midazolam on management of status epilepticus in children. Arch Pediatr. 2021;28(8):696-701.
34. Mehta V, Singhi P, Singhi S. Intravenous sodium valproate versus diazepam infusion for the control of refractory status epilepticus in children: a randomized controlled trial. J Child Neurol. 2007;22(10):1191-7.
35. van Gestel JP, Blusse van Oud-Alblas HJ, Malingre M, Ververs FF, Braun KP, van Nieuwenhuizen O. Propofol and thiopental for refractory status epilepticus in children. Neurology. 2005;65(4):591-2.
36. Misra UK, Kalita J. A comparison of four antiepileptic drugs in status epilepticus: experience from India. Int J Neurosci. 2016;126(11):1013-9.
37. Ngampoopun M, Suwanpakdee P, Jaisupa N, Nabangchang C. Effectiveness and Adverse Effect of Intravenous Lacosamide in Nonconvulsive Status Epilepticus and Acute Repetitive Seizures in Children. Neurol Res Int. 2018;2018:8432859.
38. Vasquez A, Farias-Moeller R, Tatum W. Pediatric refractory and super-refractory status epilepticus. Seizure. 2019;68:62-71.
39. Capovilla G, Beccaria F, Beghi E, Minicucci F, Sartori S, Vecchi M. Treatment of convulsive status epilepticus in childhood: recommendations of the Italian League Against Epilepsy. Epilepsia. 2013;54 Suppl 7:23-34.
40. Kim SJ, Lee DY, Kim JS. Neurologic outcomes of pediatric epileptic patients with pentobarbital coma. Pediatr Neurol. 2001;25(3):217-20.
41. Tasker RC, Vitali SH. Continuous infusion, general anesthesia and other intensive care treatment for uncontrolled status epilepticus. Curr Opin Pediatr. 2014;26(6):682-9.
42. Rosati A, L'Erario M, Ilvento L, Cecchi C, Pisano T, Mirabile L, et al. Efficacy and safety of ketamine in refractory status epilepticus in children. Neurology. 2012;79(24):2355-8.
43. Hofler J, Rohracher A, Kalss G, Zimmermann G, Dobesberger J, Pilz G, et al. (S)-Ketamine in Refractory and Super-Refractory Status Epilepticus: A Retrospective Study. CNS Drugs. 2016;30(9):869-76.
44. Lee WK, Liu KT, Young BW. Very-high-dose phenobarbital for childhood refractory status epilepticus. Pediatr Neurol. 2006;34(1):63-5.
45. Tiamkao S, Mayurasakorn N, Suko P, Jitpimolmard S, Arunpongpaisal S, Phuttharak W, et al. Very high dose phenobarbital for refractory status epilepticus. J Med Assoc Thai. 2007;90(12):2597-600.
46. Crawford TO, Mitchell WG, Fishman LS, Snodgrass SR. Very-high-dose phenobarbital for refractory status epilepticus in children. Neurology. 1988;38(7):1035-40.
